# Changes in the anterior cingulate cortex in Crohn’s disease: A neuroimaging perspective

**DOI:** 10.1002/brb3.2003

**Published:** 2020-12-12

**Authors:** Ning Kong, Chen Gao, Maosheng Xu, Xuning Gao

**Affiliations:** ^1^ The First Clinical Medical College of Zhejiang Chinese Medical University Hangzhou China; ^2^ Department of Radiology The First Affiliated Hospital of Zhejiang Chinese Medical University Hangzhou China

**Keywords:** anterior cingulate cortex, brain–gut axis, Crohn's disease, microbiota, neuroimaging

## Abstract

**Introduction:**

Evidence suggests that Crohn's disease (CD) pathophysiology goes beyond the gastrointestinal tract and is also strongly associated with the brain. In particular, the anterior cingulate cortex (ACC), which plays an integral role in the first brain as part of the default mode network (DMN) and pain matrix, shows abnormalities using multiple neuroimaging modalities. This review summarizes nine related studies that investigated changes in the ACC using structural magnetic resonance imaging, resting‐state functional magnetic resonance imaging, and magnetic resonance spectroscopy.

**Methods:**

An extensive PubMed literature search was conducted from 1980 to August 2020. In a review of the articles identified, particular attention was paid to analysis methods, technical protocol characteristics, and specific changes in the ACC.

**Results:**

In terms of morphology, a decrease in gray matter volume and cortical thickness was observed along with an increase in local gyrification index. In terms of function, functional connectivity (FC) within the DMN was increased. FC between the ACC and the amygdala was decreased. Higher amplitudes of low‐frequency fluctuation and graph theory results, including connectivity strength, clustering coefficient, and local efficiency, were detected. In terms of neurotransmitter changes, the concentrations of glutamate increased along with a decrease in gamma‐aminobutyric acid, providing a rational explanation for abdominal pain. These changes may be attributed to stress, pain, and negative emotions, as well as changes in gut microbiota.

**Conclusions:**

For patients with CD, the ACC demonstrates structural, functional, and metabolic changes. In terms of clinical findings, the ACC plays an important role in the onset of depression/anxiety and abdominal pain. Therefore, successful modulation of this pathway may guide treatment.

## Graphical abstract

This review has summarized related studies that investigated changes in the ACC in using structural magnetic resonance imaging (sMRI), functional magnetic resonance imaging (fMRI), and magnetic resonance spectrum (MRS).

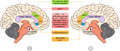

## 1 INTRODUCTION

Crohn's disease (CD) is a disabling disease affecting the gastrointestinal (GI) tract. CD is characterized by repetitive periods of remission and recurrence, and quality of life for patients with CD is significantly decreased, leading to personal and economic burden through loss of productivity (Le Berre et al., 2020). The global incidence of CD is increasing rapidly in newly industrialized regions, such as Asia and Latin America, requiring personalized medication and further investigation (Ananthakrishnan et al., 2020). Numerous studies (Agostini et al., 2017; Bao et al., 2016; Zikou et al., 2014) have detected brain anomalies using neuroimaging methods in patients with CD. The anterior cingulate cortex (ACC) is a component of the limbic system, an anatomical region of the ventromedial frontal cortex that lies superior to the corpus callosum. As an important component both in the salience network and the default mode network (Tsai et al., 2020; Tu et al., 2020), it is without doubt that the ACC has become a hot spot for functional neuroimaging and a promising candidate in CD. A comprehensive overview of changes in the ACC induced by CD pathophysiology would provide valuable insight into CD development and treatment.

As a systemic condition, the symptoms of CD are diverse. Common symptoms include abdominal pain, diarrhea, and weight loss; however, approximately half of patients also experience symptoms beyond the GI tract, including erythema nodosum or arthritis in large joints and severe fatigue (Torres et al., 2017). Extraintestinal symptoms in the brain, such as cerebral thrombosis and multiple sclerosis in white matter, have been detected (Benjilali et al., 2011; Stovicek et al., 2014), suggesting that the GI tract might have peculiar effects on the brain, despite the remote distance. Furthermore, ulcerative colitis (a subtype of inflammatory bowel disease) (Fan et al., 2019) and other brain–gut axis‐related diseases, such as irritable bowel disease (Wang et al., 2017) and functional constipation (Liu et al., 2020), show anomalies in the ACC. Thus, we hypothesize that the ACC plays an important role in the pathophysiology of CD.

The ACC has undefined boundaries and can be divided into three subregions, including the subgenual, rostral, and dorsal ACC (Tang et al., 2019). These regions interact with multiple other cortical regions, such as the primary and secondary motor cortex, orbitofrontal cortex, dorsolateral cortex, and insula (Gasquoine, 2013). The ACC functions as a relay hub, transmitting various input signals after evaluating requirements from other regions to guide adaptive behaviors. As such, the functions of the ACC range from conflict monitoring to social decision‐making, along with processing of pain and other high‐order emotions (Lichenstein et al., 2016; Xiao & Zhang, 2018). Therefore, ACC dysfunction can lead to a range of complaints, including visceral pain and depression (Li et al., 2019; Rolls et al., 2019), which are also symptoms of CD.

When homeostasis is maintained, a variety of visceral signals from the GI tract are transmitted along the extrinsic and intrinsic afferents and projected to brain regions responsible for adapting to stimuli. For example, gut‐related interoceptive signals can first be projected to the nucleus tractus solitarius and subsequently ascend the parabrachial nucleus in the brain stem, eventually reaching the insula and related networks, including the ACC (Mayer, 2011). Since the role of the ACC is associated with input signal processing and its functions are congruent with the primary clinical symptoms of CD, the role of the ACC in CD deserves deeper understanding.

## 2 MATERIALS AND METHODS

An extensive PubMed literature search was conducted from 1980 to August 2020. The following terms were used in the search: “Crohn's disease,” “brain,” “anterior cingulate cortex,” “magnetic resonance imaging,” and “magnetic resonance spectroscopy,” in all possible combinations. The search was limited to articles published in the English language. Our literature search yielded 11 potentially relevant studies. After reviewing full texts, two studies were excluded because (a) patients in the study received interventional treatment, including electro‐acupuncture or moxibustion or (b) the functional magnetic resonance imaging (fMRI) study design was based on task activation (verbal fluency task) rather than resting‐state activity. The reference lists of the selected articles were also reviewed for additional pertinent articles. When reviewing identified articles, particular attention was paid to analysis methods; the technical characteristics of structural magnetic resonance imaging (sMRI), fMRI, and magnetic resonance spectroscopy (MRS) protocols; and specific changes in, and functions of, the ACC.

## 3 RESULTS

Nine studies were included in the present review (Table 1). The retrieved studies demonstrated morphological abnormalities on sMRI, functional changes on fMRI, and concentration alterations in metabolites on MRS within ACC.

**TABLE 1:** Summary of methods and results

Ref/Year	Patients/HCs	Analysis Methods	TR (ms)	TE (ms)	Sequence^a^	ACC results	Referred function
Agostini (2012)	18/18	VBM	9.9	4.6	SPGR	GMV of left sACC negatively correlated with disease duration	Process of pain
Bao (2015)	45/35	VBM CSM	2,300	2.98	MPRAGE	Bilateral ACC’s GMV↓ Left rACC’s cortical thickness↓	Maintain positive emotions
Thomann (2016)	15/15	CSM	1900	2.13	MPRAGE	With EIMs: LGI of right rACC↑	Process of pain and sadness
Bao (2017)	47/30	VBM	2,300	2.98	MPRAGE	With abdominal pain: lower GMV in right ACC	Process of visceral pain
Thomann (2017)	15/14	FC	2,210	23	EPI	FC↑	Self‐referential
Bao (2018)	40/60	ALFF	2000	30	EPI	ALFF↑	Process of pain Monitoring
Liu (2018)	43/37	Graph theory	2000	30	EPI	With higher HADS: connectivity strength ↑ clustering coefficient ↑ local efficiency↑	Negative effects in chronic pain
Fan (2019)	42/35	FC	2000	30	EPI	FC↓between dACC and the amygdala	Process of visceral pain
Lv (2018)	29/20	/	2000 2000	35 60	PRESS MEGA‐PRESS	With abdominal pain: Glu/ tCr ↑ GABA+/ tCr ↓ lower levels of GABA+/tCr	Process of pain

Abbreviations: ACC, anterior cingulate cortex; ALFF, amplitude of low‐frequency fluctuation; CSM, cortical surface model; dACC, dorsal anterior cingulate cortex; EIMs, extraintestinal manifestations; EPI, gradient–recalled echo planar imaging; FC, functional connectivity; GABA+, aminobutyric acid plus; Glu, Glutamate; GMV, gray matter volume; HADS, hospital Anxiety and Depression Scale; HCs, healthy controls; LGI, local gyrification index; MEGA‐PRESS, Mesher‐Garwood point‐resolved spectroscopy; MPRAGE, magnetization prepared rapid gradient echo;rACC, rostral anterior cingulate cortex; sACC, subgenual anterior cingulate cortex; SPGR, spoiled gradient recalled echo; tCr, total creatine; TE, echo time; TR, repetition time; VBM, voxel‐based morphometry.

^a^ All the imaging data were collected using 3‐Tesla MRI scanner.

### 3.1 Neuroimaging of the ACC in CD patients

**FIGURE 1:**
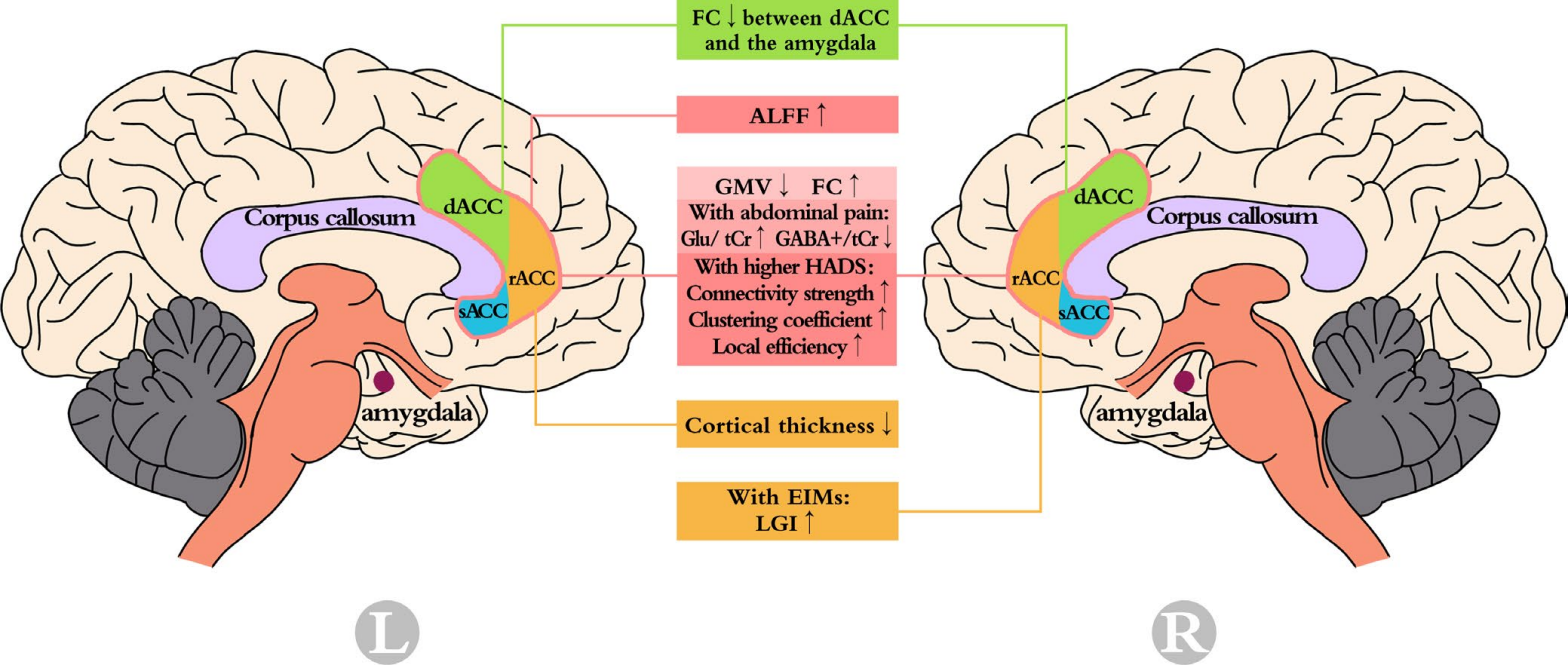
Anterior cingulate cortex changes in patients with Crohn's Disease detected by neuroimaging

Neuroimaging is a valuable tool that can be used to probe localized regions within the brain to examine the microstructure and interactions in vivo using a noninvasive, real‐time method. Integrating image data with clinical measurements also aids the understanding of specific pathophysiological processes. Nowadays, prevalent modalities can be divided into three groups. The first demonstrates morphological alterations, including sMRI and diffusion tensor imaging, which mainly focus on gray matter (GM) and white matter (WM), respectively. The second type of functional modality includes fMRI and electroencephalography (Sui et al., 2014). The third method reflects metabolic changes and mainly relies on MRS. Neuroimaging has been widely utilized to examine neurological diseases and psychiatric disorders. Changes in the ACC in patients with CD are shown (Figure 1).

### 3.2 Anomalies in the ACC with structural neuroimaging

High‐resolution T1‐weighted imaging is an indispensable approach for examining anatomical information using sMRI, and two orientations exist for image analysis. The first, which is more commonly used, is volume‐based analysis, among which voxel‐based morphometry (VBM) is the more practicable approach. VBM enables segmentation of different tissue types, such as GM, WM, and cerebrospinal fluid, with reliable intergroup differentiation (Kanai & Rees, 2011). The second method of analysis is based on surface properties; for instance, a cortical surface model (CSM) that accentuates the cortical surface area, thickness, and folding. From this information, the GM volume can be determined by taking the product of the first two parameters (Winkler et al., 2010).

For most individuals diagnosed with CD, abdominal pain is a persistent and common symptom. Agostini's study (2013) was the first to focus on pain matrix structural changes in patients with CD, shedding light on morphological studies based on the brain–gut axis (BGA) theory. This study utilized VBM to distinguish between altered brain regions after comparing 18 patients in remission to matched healthy controls (HCs). The GM volume of the subgenual ACC was negatively correlated with disease duration. As a result, a smaller GM volume corresponded to a greater disease burden, highlighting the cytotoxic influence of inflammatory mediators exerted on neurons and bottom‐up transmission from the GI tract, which results in neural atrophy and GM loss.

To further identify the specific brain regions affected by CD, Bao et al. (2015) enlarged the sample size to 45 and combined measurements of GM volume and cortical thickness to strengthen the data. The findings revealed additional affected regions and revealed both GM loss and GM gain. One of their innovations was use of the Hospital Anxiety Depression Scale (HADS) score as a covariate to rule out psychological effects (e.g., depression, anxiety) on brain changes and simultaneously imply their contributions to plasticity. This was also the first investigation to examine changes in cortical thickness in patients with CD. ACC results demonstrate that GM volume was decreased in bilateral ACC, and GM volume loss in the right ACC was negatively correlated with disease duration. The cortical thickness in the rostral ACC was also decreased. Consistent with their hypothesis, when considering the HADS scores, differences in GM volume clusters and cortical thickness were not as marked as suggested, indicating the role of psychiatric disorders on brain morphology and involvement of the ACC in emotional processing.

With regard to abdominal pain in patients with CD, Bao's VBM analysis (Bao et al., 2017) further related aberrant pain signal transmission and pain‐related emotion with the ACC. They discovered that the GM volume of the insula and right ACC in patients with CD with abdominal pain was reduced compared with HCs and those with CD without abdominal pain. This finding was congruent with their previous study (Bao et al., 2015) and studies that focused on the relationship between ACC and other chronic pain disorders, such as irritable bowel syndrome (Qi et al., 2016). As mentioned above, symptoms of CD include extraintestinal complaints (e.g., arthritis, erythema nodosum) in addition to localized abdominal disorders. It is plausible that patients with extraintestinal manifestations (EIMs) are more vulnerable to aberrant brain involvement. Thomann et al. (2016) utilized the CSM method and revealed that the local gyrification index of the right rostral ACC in patients with EIMs was larger compared with those without EIMs. This indicates that in the former subgroup, the amount of cortex buried below the sulcus fold is greater. Since GM volume is more often employed in research, gyrification might be used as another specific signature of CD. It might even be used as a biomarker for predicting CD progress combined with other parameters, since neurological abnormalities may precede disease onset (Heinze et al., 2020).

### 3.3 Anomalies in the ACC with functional neuroimaging

Use of blood‐oxygen‐level‐dependent imaging, which detects hemodynamic responses to alterations in neural metabolism or spontaneous fluctuations, forms the basic premise of fMRI. By responding to local neuronal firing rates, fMRI is able to discern resting‐state dynamics, wherein the subject is not engaged in an active task. To analyze such data, seed‐based analysis and independent component analysis (ICA) are widely used to determine functional connectivity (FC). Graph theory models the brain's topology and functional organization by counting a network as a set of nodes and edges, representing the network's elements and intergroup relationships (Chen & Glover, 2015; Sui et al., 2014). With the resting‐state approach, regions with temporal correspondence are extracted, comprising resting‐state networks, such as the default mode network (DMN) and the salience network (Barkhof et al., 2014). Methods derived from fMRI, such as the amplitude of low‐frequency fluctuation (ALFF) (Yang et al., 2007) and regional homogeneity (Zang et al., 2004), can also be used to evaluate spontaneous neural activity, other than FC.

Thomann et al. (Thomann et al., 2017) examined the role of the DMN in patients with CD due to its role in various psychiatric disorders, such as depression and anxiety (Coutinho et al., 2016), which affect numerous patients with CD (Clark et al., 2014; Reigada et al., 2015). By comparing 15 patients with CD in remission against 14 HCs, disruption to the functional integrity of the DMN was noted. Within the anterior part of the DMN, a cluster comprising the ACC exhibited increased FC, though no significant correlations were observed between it and other clinical variables, such as depression and fatigue. It can be extrapolated that the constant relapsing/remitting nature of CD can have a long‐term impact on mental status, which may manifest as changes in the ACC, leading to anxiety and depression.

In addition to changes in the DMN subsystem, Fan et al. (Fan et al., 2020) found that FC between the amygdala and the dorsal ACC was decreased in 42 CD patients compared with 35 HCs. Their finding suggests a decreased ability to mediate visceral sensation. Building upon the evidence that long‐term inflammation causes alterations in the brain (Kim et al., 2019) and may impact functional results, Bao et al. (2018) used other methods, including ALFF and FC, to assess anomalies in the intrinsic activity of the brain after scanning 60 patients with CD. Their study revealed increase in ALFF value in the ACC, further highlighting the role of the ACC in CD symptoms, such as visceral pain.

In addition to the analysis methods mentioned above, graph theory is another data‐driven approach used to explore the topological conditions of intrinsic connectivity networks (ICNs) and data extracted from fMRI processed with ICA. In Liu's report (2018), CD patients with a higher HADS score demonstrated an increase in connectivity strength, clustering coefficient, and local efficiency in the ACC. These findings suggest an impairment in the “small world” pattern, an aberrant ability to transform and integrate information and emotional regulation, and are consistent with previous studies suggesting a role for the ACC in psychiatric disorders.

It is worth mentioning that for these aforementioned fMRI studies, an important point has been overlooked. In most cases, patients with CD undergo administration of contrast agent for abdominopelvic MRI to differentiate active from quiescent disease, which makes them more prone to gadolinium accumulation in brain. Though Mallio et al. (2019, 2020) had revealed that the dentate nucleus where gadolinium predominantly accumulates did not show significant functional connectivity changes, it shed light on us that we should be more careful on the research design and consider those clinical factors that relate closely to CD patients.

### 3.4 Anomalies in the ACC with MRS

^1^H‐MRS is the sole imaging method used to quantify localized metabolites or neurotransmitters, such as glutamine, glutamate (Glu), and gamma‐aminobutyric acid (GABA) (Squarcina et al., 2017). The results are often expressed as ratios relative to creatine (Jansen et al., 2006). With regard to CD, MRS has been used to examine the colonic mucosa ex vivo and was found to be useful in the diagnosis of CD (Bezabeh et al., 2001). Lv et al. (2018) was the first to apply MRS in CD patients in vivo to examine changes in metabolites and neurotransmitters in bilateral ACC and their correlation with abdominal pain. This study demonstrated that compared with HCs, CD patients with significant pain had higher Glu/tCr and lower GABA/tCr values. The former was positively correlated with visual analogue scale scores and the latter acted inversely with the Crohn's disease activity index. Since Glu and GABA are the principal excitatory and inhibitory neurotransmitters in the central nervous system, respectively, they are critical mediators in pain conditions, such as chronic pelvic pain (Harper et al., 2017), as well as psychiatric conditions, such as depression (Gabbay et al., 2017). Therefore, the ACC serves a crucial role as both a pain‐processing and an emotion‐processing region in patients with CD.

## 4 DISCUSSION

### 4.1 Brain and gut: bidirectional communication

The cause and pathophysiology of CD have been investigated from multiple angles, including genetic, environmental, immune (abnormal responses caused by intestinal barrier function or innate immune defects), and microbial perspectives (Torres et al., 2017). In recent years, the focus has shifted primarily to the role of the invisible axis between the gut (down) and the brain (top), which is a bidirectional pathway comprised of the central nervous system, autonomic nervous system, enteric nervous system, and hypothalamic–pituitary–adrenal (HPA) axis (Raskov et al., 2016). This pathway contributes to the psychiatric comorbidities of CD, including depression and anxiety (Gracie et al., 2018), and development of abdominal pain syndromes (Mayer & Tillisch, 2011). Activation of the HPA axis is a classic example of top‐down control. In psychological disorders, increases in adrenocorticotropic hormone release, which increases intestinal permeability, could exacerbate the course of CD (Gracie et al., 2019). Conversely, from a down‐top perspective, in which the gut can influence the brain, microbiota in the gut play a key role and have become increasingly important in recent years; the relationship has even been renamed as the “microbiota–gut–brain axis” to highlight its effects on gut–brain pathways. The unique composition of microbial flora is also considered one of the etiologies of CD, which can be affected by both intrinsic and extrinsic factors. The former includes personal genetic susceptibility, hormonal and immune ability, and regulation by the central nervous system. The latter relates more to one's lifestyle; diet, use of medications, and personal habits all play a key role. Under physiological conditions, a symbiotic relationship between the host and microbiota is maintained. However, once the composition of microbiota changes, profound effects occur in the brain (Zhu et al., 2020). Therefore, it is clear that gut microbiota and the bidirectional axis are associated with the pathogenesis and manifestation of CD. Aside from CD, changes in gut microbiota are also broadly reported in neurological diseases, including Alzheimer's disease, Parkinson's disease, and autism spectrum disorders, further proving the interaction between the brain and gut (Osadchiy et al., 2019).

### 4.2 Stress, pain, negative emotions, and microbiota as indicators

**FIGURE 2:**
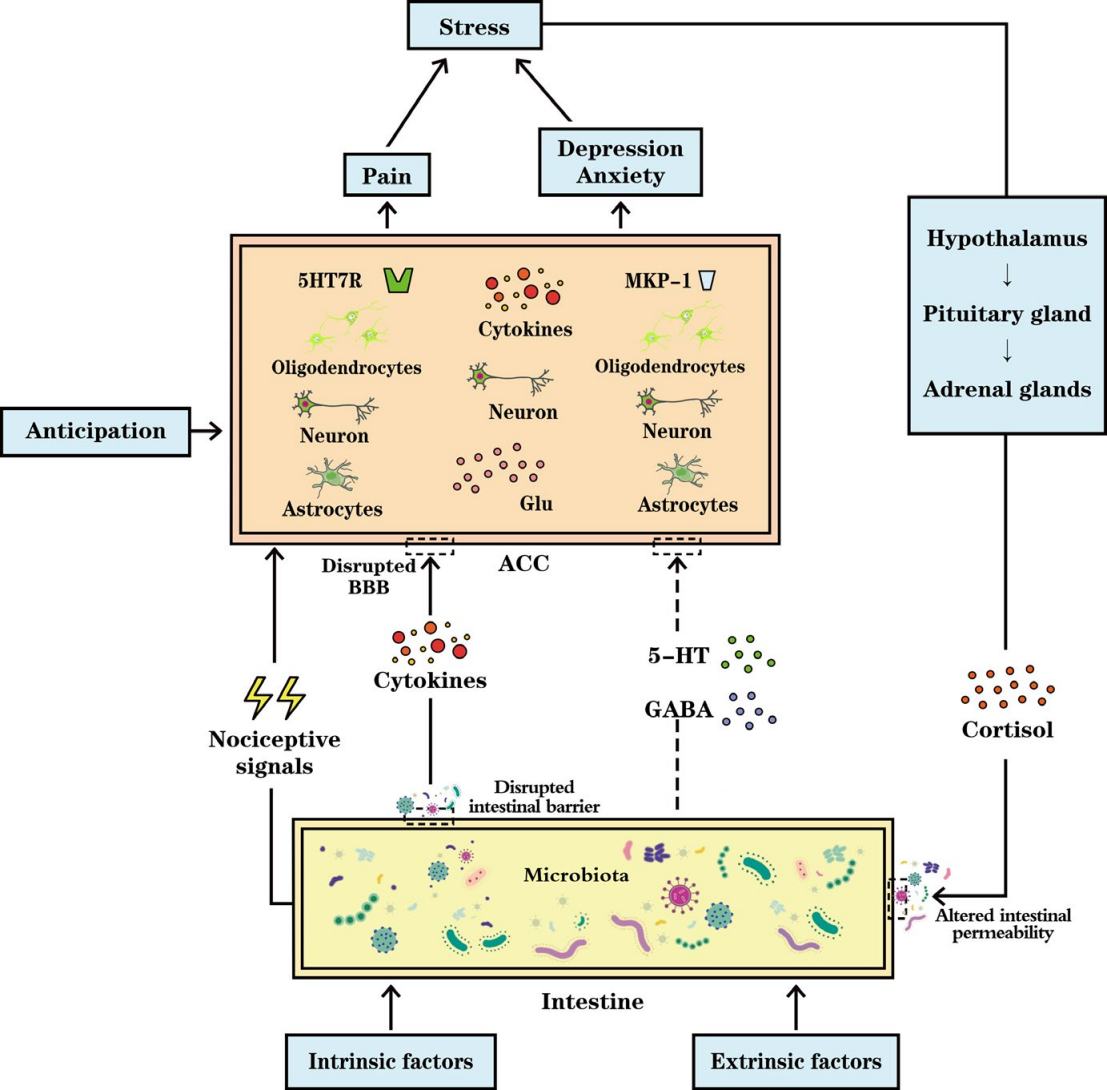
The anterior cingulate cortex (ACC) undergoes long‐term potentiation by processing nociceptive signals, and excessive glutamate (Glu) release may lead to neuronal injury. Overexpression of mitogen‐activated kinase phosphatase‐1 (MKP‐1) in the ACC has been implicated to trigger depression. The uncertainty and anticipation in patients with CD may induce anxiety and lead to ACC activation. Symptoms aggravate stress, activating the hypothalamic–pituitary–adrenal axis, secreting cortisol, and increasing intestinal permeability. Gut microbiota, the composition of which is influenced by both intrinsic and extrinsic factors, regulate intestinal barrier function. In case of dysbiosis, bacteria and their products can cross the barrier, eliciting inflammatory cytokine release and increasing blood–brain barrier (BBB) permeability. Circulating cytokines cross the BBB, inducing astrocyte and oligodendrocyte apoptosis, further disrupting BBB integrity. It is still to be ascertained whether gamma‐aminobutyric acid (GABA) and 5‐HT generated by microbiota can cross the disrupted BBB of patients with CD, for 5HT7R has a marked impact on brain remodeling

Acting as a relay to convey information from many other brain regions, the ACC is a hub that orchestrates multiple signals, manipulating the functions of emotional behavior and cognition. Based on the bidirectional pathway, the underlying mechanism of the changes is intricate (Figure 2). For instance, a review by Vogt (2013) discussed that pain may contribute to structural changes in the ACC. Gut inflammation and stress evoke cingulate‐mediated psychiatric illnesses, such as depression. However, with in‐depth study, more factors, such as microbiota, brain metabolites, and the influence of negative emotions, should be also considered.

Since CD is progressive and relapsing, it would cause a substantial quality‐of‐life burden, putting patients under great stress. Subsequent symptoms, including abdominal pain, and negative emotions, such as depression and anxiety, exacerbate this stress. Being the prominent symptom, chronic pain contributes greatly to the observed changes. As a part of the “pain matrix,” the ACC processes information transmitted by visceral nociceptive afferents and undergoes long‐term potentiation, which affects excitatory synapses and results in cortical plasticity (Zhuo, 2020). Rosso et al. (2017) suggested that hippocampal atrophy in posttraumatic stress disorder partly reflects stress‐induced Glu excitotoxicity, which can culminate in neuronal injury. Since Lv's research (Lv et al., 2018) revealed that CD patients with pain exhibit higher Glu concentrations in the ACC and the excitotoxic effect of this crucial excitatory transmitter is widely known (Hernández et al., 2018), the influence of excessive Glu on cell death and synaptic loss in the ACC are of potential concern (Jung et al., 2019). Depression is a common psychological comorbidity in patients with CD (Filipovic & Filipovic, 2014), which is associated with the ACC (Lichenstein et al., 2016; Rolls et al., 2019). It is noteworthy that in Di's research, overexpression of mitogen‐activated kinase phosphatase‐1 in the ACC was implicated as a trigger for the pathogenesis of depression (Di Benedetto, 2017). In addition, Rubio et al. suggested that the uncertainty and anticipation of the disease's relapse patterns may induce anxiety, leading to activation of the ACC (Rubio et al., 2015). As mentioned before, neurotransmitter activity is the basis of relative blood oxygenation and blood‐oxygen‐level‐dependent fMRI; therefore, abnormalities in the ACC with MRS could partly explain the functional changes mentioned above. As symptoms aggravate stress, the HPA axis would secrete more cortisol to the intestine, increasing its permeability, which is a vicious cycle.

Gut microbiota, the composition of which is influenced by both intrinsic and extrinsic factors, significantly affects CD pathogenesis. Microbiota affect pain sensation (Clarke et al., 2012) and generate neurotransmitters, such as GABA and 5‐hydroxytryptamine (5‐HT) (Barrett et al., 2012; Lyte, 2011). 5‐HT is critical in the BGA pathway, and its receptors (5HT7R) on dendritic cells play a key role in synaptic modulation and neuronal circuit reorganization, which lead to brain remodeling and morphological changes (Crispino et al., 2020). Gut microbiota have a crucial role in the functionality of innate and adaptive immune responses and intestinal barrier homeostasis (Cryan & Dinan, 2012). Under normal conditions, gut microbiota restrict commensal‐related inflammatory responses and maintain equilibrium between tolerance and immunity to antigens to regulate intestinal barrier function. However, in cases of dysbiosis, bacteria and their products cross the barrier, eliciting inflammatory cytokine release (Vuotto et al., 2020). Like the intestine, brain parenchyma is also protected by a barrier, the blood–brain barrier (BBB), which prevents toxins and pathogens from entering and regulates molecular transport between the periphery and central nervous system. However, circulating cytokines can disrupt BBB integrity and increase its permeability, leaving the brain vulnerable to cytokines from gut‐derived bacteria (Abautret‐Daly et al., 2018). Cytokines, such as tumor necrosis factor alpha cross the BBB, propagating inflammatory signals by activating glial cells. Cytokines may modulate neural plasticity by inducing astrocyte and oligodendrocyte apoptosis, which decreases neurogenesis (Miller et al., 2009). Since astrocytes are indispensable in maintaining the stability of the BBB, the barrier's permeability is further disrupted. It is noteworthy that the ACC is particularly sensitive to cytokines (Miller et al., 2013), which may help to explain its involvement in inflammatory CD. Peripheral molecules, such as 5‐HT and GABA, do not typically cross the BBB. However, BBB disruption in CD may allow their passage, so it is still to be ascertained whether changes in barrier permeability induced by diverse CD factors allow them into the central nervous system. Unsolved abnormal MRS results and brain morphological changes can be further explained if such transit exists.

### 4.3 Clinical symptoms and treating the ACC in CD

According to research on changes in the ACC in patients with CD, abdominal pain, and psychiatric disorders, including depression and anxiety, are major symptoms. Although it has not been confirmed as a specific treatment target for CD, we hypothesize that modulating activity in the ACC may help alleviate the common complaints of patients with CD. For instance, deep brain stimulation for relief of pain is being investigated, and the dorsal ACC has been considered another potential target for neuromodulation (Russo & Sheth, 2015). Ketamine has shown great antidepressant efficiency by reducing the hyperactivity of the subgenual ACC (Morris et al., 2020), and noninvasive intermittent theta‐burst stimulation is also helpful in relieving depression by changing the FC of the subgenual ACC (Cole et al., 2020).

Until now, only nine studies have reported ACC abnormalities in patients with CD. A recent neuroimaging meta‐analysis on brain changes in patients with CD using 16 original studies did not find a consistent association between the ACC and CD (Yeung, 2020). This might be due to inclusion of studies with different designs and populations; thus, the ACC did not stand out from pooled heterogenous data. Therefore, more studies are needed to further augment our current understanding.

Another challenge in the study of CD is the bidirectional characteristics of the BGA, which make it difficult to discern whether clinical findings are a cause or consequence of changes in the ACC. We agree that they supplement each other, and future studies with larger sample sizes would provide more statistical power. As described earlier, the functions of ACC subregions vary. The subgenual ACC is responsible for depression, while the dorsal ACC is responsible for pain. Therefore, future studies on the ACC should utilize segmentation and refine findings to the dorsal, rostral, and subgenual regions along with multimodal methods, such as positron emission tomography‐MRI, to examine metabolic environments.

## 5 CONCLUSIONS

The ACC serves a pivotal role in CD according to neuroimaging. In alignment with the brain–gut axis, CD symptoms, such as pain, depression, and anxiety, may be the result of disruption along this bidirectional pathway. As such, targeting the ACC for treatment may help alleviate the common symptoms of CD. Future studies should examine the potential of this approach. Despite the promising findings, ACC research is still in its infancy; thus, further studies are necessary.

## CONFLICT OF INTEREST

There is no conflict of interest to be disclosed.

## AUTHOR CONTRIBUTIONS

Ning Kong and Chen Gao drafted and formatted the manuscript. Ning Kong created figures. Maosheng Xu and Xuning Gao provided supervision and edited the final manuscript and should be considered joint senior author. All authors approved the final manuscript as submitted.

### Peer Review

The peer review history for this article is available at https://publons.com/publon/10.1002/brb3.2003.

## Data Availability

Data sharing is not applicable to this article as no new data were created or analyzed in this study.
